# Supplementary material for: Causal Relationship between Aging and Anorexia Nervosa: A White-Matter-Microstructure-Mediated Mendelian Randomization Analysis
Source: Biomedicines. 2024 Aug 16;12(8):1874. doi: 10.3390/biomedicines12081874 (PMC11351342; doi:10.3390/biomedicines12081874)
Supplement: Supplementary file 1 [file biomedicines-12-01874-s001.zip › Supplementary Figure/Figure S4/Singe SNP plot of MR analysis for TL to ubm-b-1927.pdf]

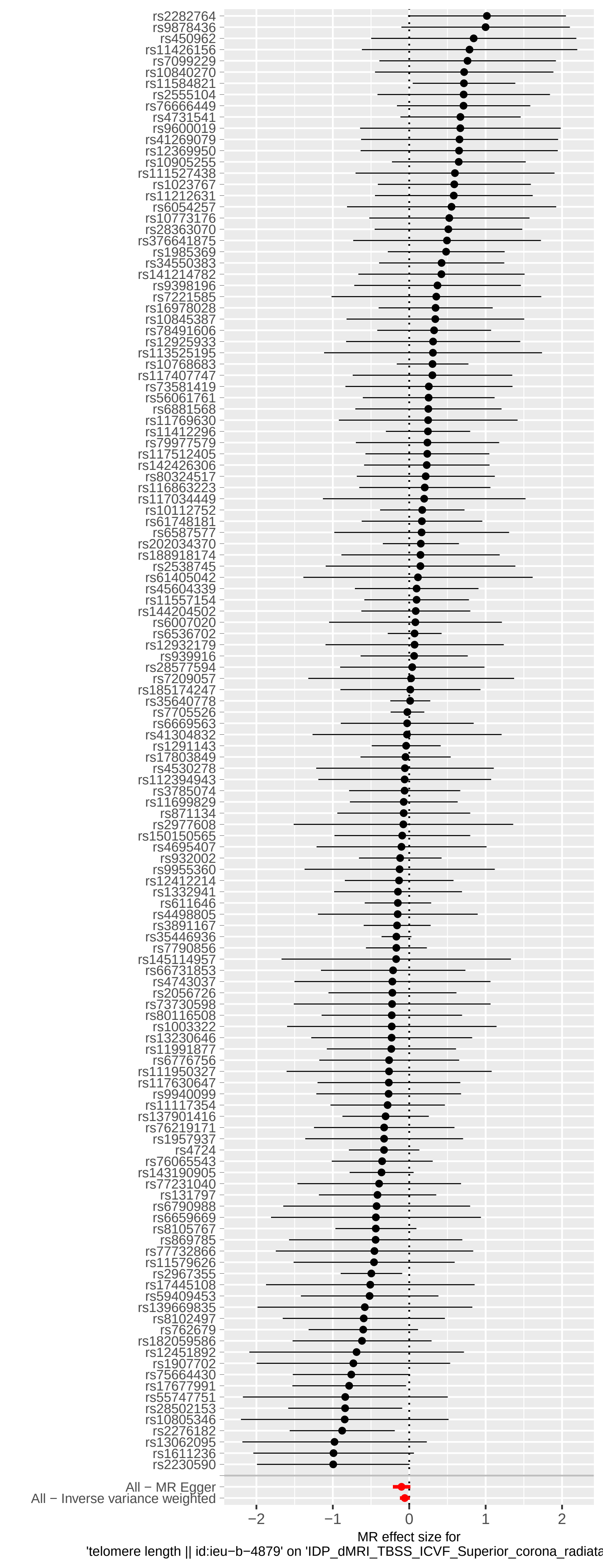
rs2282764
rs9878436
rs450962
rs11426156
rs7099229
rs10840270
rs11584821
rs2555104
rs76666449
rs4731541
rs9600019
rs41269079
rs12369950
rs10905255
rs111527438
rs1023767
rs11212631
rs6054257
rs10773176
rs28363070
rs376641875
rs1985369
rs34550383
rs141214782
rs9398196
rs7221585
rs16978028
rs10845387
rs78491606
rs12925933
rs113525195
rs10768683
rs117407747
rs73581419
rs56061761
rs6881568
rs11769630
rs11412296
rs79977579
rs117512405
rs142426306
rs80324517
rs116863223
rs117034449
rs10112752
rs61748181
rs6587577
rs202034370
rs188918174
rs2538745
rs61405042
rs45604339
rs11557154
rs144204502
rs6007020
rs6536702
rs12932179
rs939916
rs28577594
rs7209057
rs185174247
rs35640778
rs7705526
rs6669563
rs41304832
rs1291143
rs17803849
rs4530278
rs112394943
rs3785074
rs11699829
rs871134
rs2977608
rs150150565
rs4695407
rs932002
rs9955360
rs12412214
rs1332941
rs611646
rs4498805
rs3891167
rs35446936
rs7790856
rs145114957
rs66731853
rs4743037
rs2056726
rs73730598
rs80116508
rs1003322
rs13230646
rs11991877
rs6776756
rs111950327
rs117630647
rs9940099
rs11117354
rs137901416
rs76219171
rs1957937
rs4724
rs76065543
rs143190905
rs77231040
rs131797
rs6790988
rs6659669
rs8105767
rs869785
rs77732866
rs11579626
rs2967355
rs17445108
rs59409453
rs139669835
rs8102497
rs762679
rs182059586
rs12451892
rs1907702
rs75664430
rs17677991
rs55747751
rs28502153
rs10805346
rs2276182
rs13062095
rs1611236
rs2230590
All − MR Egger
All − Inverse variance weighted
−2
−1
0
1
2
MR effect size for
'telomere length || id:ieu−b−4879' on 'IDP_dMRI_TBSS_ICVF_Superior_corona_radiata
